# Microphysiological systems

**DOI:** 10.1063/1.5130170

**Published:** 2019-10-29

**Authors:** James J. Hickman, Dongeun Huh, Roger D. Kamm

**Affiliations:** 1 NanoScience Technology Center, University of Central Florida, 12424 Research Parkway Suite 400, Orlando, Florida 32826, USA; 2 Department of Bioengineering, University of Pennsylvania, Philadelphia, Pennsylvania 19104, USA; 3 Department of Biological Engineering and Department of Mechanical Engineering, Massachusetts Institute of Technology, 500 Technology Square, MIT Building, Room NE47-321, Cambridge, Massachusetts 02139, USA

## INTRODUCTION

In the special topic, “Microphysiological Systems,” published in *APL Bioengineering,* we are pleased to present a special collection of papers that provide a window into the microfluidic platform technologies that are now being developed to simulate and study a variety of processes in mammalian biology/physiology and human disease, with important applications in preclinical screening. Historically, microfluidics began its steep ascent following a 2005 paper by the group of George Whitesides who introduced the concept of using polydimethylsiloxane (PDMS) to quickly and easily fabricate microscale systems (micrometers to millimeter) with multiple channels or chambers.^1^ More recently, researchers found that with the seeding of different cell types, either on 2-dimensional (2D) surfaces or in 3-dimensional (3D) matrices, they could recapitulate certain key biological processes, leading to a second wave in which “organ-on-chip” systems soon emerged, of progressively increasing realism and complexity.^2,3^ This field evolved into what is now commonly referred to as Microphysiological Systems or “MPS,” which are finding applications not only as models of healthy biology but also as disease models and drug screening platforms. The latter developments have driven considerable interest on the part of not only the research community but also the biotech and pharmaceutical industries as they seek to identify ways of improving the overall success rates of drugs but especially those that enter clinical trials. Driven by this interest, numerous companies have been launched in order to make the platforms that enable MPS available to the wider community. Apart from the vast range of applications of MPS, they also differ in terms of the cell type used (cell lines, primary cells, and iPSC-derived), their complexity (single cell, suborgan, organ, or multiple organ systems), and their inclusion of sensors, electrodes, or sophisticated image techniques employed.

## SUMMARY OF THE AREAS COVERED

As the field evolves, one finds numerous approaches, as well as a wealth of fundamental questions and issues that can effectively be addressed, making MPS a fertile area of investigation and development. While MPS can be characterized along numerous dimensions, it is first useful to recognize that they all, to varying degrees, rely on cellular self-assembly and the emergent properties of multicellular systems. Indeed, one of the primary benefits of MPS is that they facilitate multicell interactions to produce *in vivo*-like structures (muscle, neural networks, and epithelial barriers), thus yielding deeper insights into how cells interact in such a way so as to produce highly structured systems from populations of single or multiple cells. One such case that is explored in several of the papers in this collection is the formation of vascular networks or single vessels lined with endothelial cells (vessels-on-a-chip). The challenges here are to create vessels that recapitulate both the morphology and the function of the *in vivo* vasculature. de Graaf *et al.*^4^ provide new guidance into the application of a process called viscous fingering to produce open channels in collagen type 1 gels that can subsequently be seeded with endothelial cells. By systematically varying the experimental parameters, they achieve a high success rate in forming vessels from human iPS cells.

Abe *et al.*^5^ investigate the balance between biochemical factors that induce angiogenesis and an alternative approach in which transmural flow takes place across the monolayer from the cell-extracellular matrix (ECM) into the open lumen. They find that, interestingly, either biochemical or mechanical stimuli can produce similar levels of angiogenic sprouting from an endothelial monolayer.

Understanding the relationship between MPS and the organs or organ systems, they aim to mimic often, which is critical for both validation and the interpretation of the obtained results. Sung *et al.*^6^ address an important dimension of this through a review of the various mathematical or computational methods that can be brought to bear in these situations. Of particular interest in this context is understanding the interactions between organs in a multiorgan MPS and the essential scaling issues that need to be considered to mimic these interactions *in vivo*.

Jusoh *et al.*^7^ present an organ-on-chip method to model the effect of skin irritation on growth of new vasculature. This addresses the important need for drug screening platforms that can be used to supplement or even replace animal studies in experiments of new chemicals or cosmetics, an issue with particular relevance in view of the increasing pressures for a global ban on animal testing. Through the use of three cell types, keratinocytes, dermal fibroblasts, and endothelial cells, they present proof-of-principle results for the use of the model to screen for the irritant effects of two common ingredients in cosmetics, paving the way for a broader range of applications in the future.

Muscle has been the focus of much research with MPS, and a variety of platforms have been reported to simulate different issues that arise in both skeletal and cardiac tissues. One of the problems that often arise in *in vitro* models is that the elevated rate of oxygen consumption in exercising muscle can often lead to hypoxia and necrosis of the tissue, especially in the core of larger specimens. *In vivo*, this is avoided by increased blood delivery via an extensive vascular network, but to date, few approaches have been developed for MPS that can provide the levels of gas exchange necessary to maintain oxygenated, fully functional tissues. Two papers in this collection present models that address different aspects of muscle behavior in the context of hypoxia. Davis *et al.*^8^ use a combination of experiments and transport modeling to examine the exercise-induced changes in muscle respiration *in vitro* and the ability of convective flows around the muscle to enhance oxygen delivery. They argue that previous studies may well have been negatively impacted by these effects and that methods to augment delivery should be considered in the design of new platforms. Oleaga *et al.*,^9^ however, investigate the problem of cardiac injury resulting from a transient ischemic event. These authors employ a novel device in which iPSC-derived cardiomyocytes are seeded onto a microelectrode array that enables changes in cardiac conduction velocity, beat frequency, and QT intervals to be measured and propose it as a useful tool for preclinical drug screening for cardioprotective drugs. They go on to show that the platform not only can simulate tissue damage but is also useful as a means of studying the effects of therapeutic interventions or screening for new pharmacological agents.

The scope of organ systems being explored continues to expand. Here, Brooks *et al.*^10^ present one application in the context of cancer, introducing a polyethylene glycol hydrogel system that they engineer to mimic certain aspects of the ECM of the omentum, using this material in a 3D MPS platform with tumor spheroids generated from an ovarian cancer cell line to produce new insights into the emergence of drug resistance and introducing a new platform for preclinical screening of pharmacologic interventions.

## CONCLUSIONS

In summary, the special topic, Microphysiological Systems, provides a glimpse into the rapidly expanding field of MPS research, providing new directions for current researchers, a useful introduction for those just entering the field, and a useful snapshot for those wishing to keep abreast of new developments in an exciting field.
